# Artificial Intelligence in Pediatric Electrocardiography: A Comprehensive Review

**DOI:** 10.3390/children12010025

**Published:** 2024-12-27

**Authors:** David M. Leone, Donnchadh O’Sullivan, Katia Bravo-Jaimes

**Affiliations:** 1 Cincinnati Children’s Hospital Heart Institute, University of Cincinnati, Cincinnati, OH 45229, USA; 2 Department of Pediatric Cardiology, Texas Children’s Hospital, Baylor College of Medicine, Houston, TX 77030, USA; 3 Department of Cardiovascular Medicine, Mayo Clinic, Jacksonville, FL 32224, USA

**Keywords:** artificial intelligence, electrocardiogram, machine learning, deep learning, convolutional neural networks

## Abstract

Artificial intelligence (AI) is revolutionizing healthcare by offering innovative solutions for diagnosis, treatment, and patient management. Only recently has the field of pediatric cardiology begun to explore the use of deep learning methods to analyze electrocardiogram (ECG) data, aiming to enhance diagnostic accuracy, expedite workflows, and improve patient outcomes. This review examines the current state of AI-enhanced ECG interpretation in pediatric cardiology applications, drawing insights from adult AI-ECG research given the progress in this field. It describes a broad range of AI methodologies, investigates the unique challenges inherent in pediatric ECG analysis, reviews the current state of the literature in pediatric AI-ECG, and discusses potential future directions for research and clinical practice. While AI-ECG applications have demonstrated considerable promise, widespread clinical adoption necessitates further research, rigorous validation, and careful consideration of equity, ethical, legal, and practical challenges.

## 1. Introduction

Electrocardiography (ECG) is a safe, inexpensive, and widely available diagnostic tool used around the world. Artificial intelligence (AI), particularly through deep learning (DL) and convolutional neural network (CNN) architectures, enables advanced computer vision capabilities in ECG analysis [1]. With robust performance on large clinical datasets, AI algorithms in ECG interpretation have demonstrated accurate predictive abilities through supervised learning. Since 2018, there has been rapid development and validation of ECG-based DL for the detection of several cardiovascular conditions. Algorithms using ECG data (AI-ECG) have shown capabilities ranging from accurately predicting left ventricle (LV) and right ventricle (RV) dysfunction to diagnosing conditions such as aortic stenosis, hypertrophic cardiomyopathy, cardiac amyloidosis, long QT syndrome, and atrial septal defects (ASD) [2,3,4,5,6,7,8,9,10]. These algorithms have also been able to identify patterns linked to features such as the patient’s sex, age, risk of developing atrial fibrillation, hyperkalemia, and mortality [11,12,13,14,15,16]. Many of these conditions cannot be reliably determined by a human cardiologist’s ECG interpretation.

One of the more widely studied AI-ECG algorithms in adults focuses on the detection of LV systolic dysfunction. These algorithms have achieved not only excellent performance but have maintained this accuracy when undergoing multicenter, external validation. Interestingly, these studies have also shown that those initially labeled as “false positives” have an observed elevated 5–10-year risk of later developing reduced LV function [17,18]. These algorithms are being deployed with pragmatic, randomized clinical trials and have shown improvements and cost reductions in detecting LV dysfunction across primary care and obstetric settings [16,19,20,21]. In addition, the United States Food and Drug Administration (FDA) clearance of these algorithms has made them available to clinicians at the point of care via dashboards embedded in electronic medical records, smartphone applications, and digital stethoscopes [22,23]. Although this represents an important example of AI-ECG’s potential, we acknowledge that there have been significant challenges when trying to achieve similar progress in pediatrics.

Heart disease that affects children is often quite different in etiology and scope compared to that of adults with acquired heart disease. Pediatric cardiovascular disorders predominantly include congenital heart disease (CHD), which is the most common congenital malformation and occurs in approximately 1% of live births worldwide, contributing substantially to morbidity and mortality [24,25,26]. Additionally, children rarely experience ischemic heart disease, and the incidences of arrhythmia and cardiomyopathy are much lower compared to adults. Despite these differences, early detection and accurate diagnosis remain critical for timely intervention and improved outcomes. Interpretation of the pediatric ECG (pECG) is particularly challenging due to age-dependent physiological changes, variations in normal values, and the complex spectrum of CHDs that cause diverse ECG changes [27,28]. Further limitations in this interpretation include inter-observer variability and the need for specialized expertise, which can lead to delayed or missed diagnoses. Automated AI-enabled interpretation of the pECG has the potential to overcome these limitations and improve equity in early detection, but this requires multicenter collaboration to construct diverse datasets and to achieve external generalizability.

Despite the aforementioned challenges, recent studies, such as those by Chen et al., have begun to explore the potential of AI in pECG analysis, demonstrating promising results in detecting CHDs, whereas Mayourian et al. and Anjewierdan and O’Sullivan et al. have shown promise in predicting pediatric ventricular dysfunction [3,29,30]. Additionally, insights from adult AI-ECG research, such as with wearable technologies, may offer valuable strategies applicable to pediatric populations [31,32]. This review provides a comprehensive overview of the current state of AI applications in pECG analysis. We discuss a broad range of AI techniques used, explore the challenges specific to pECG interpretation, and critically analyze key findings from recent studies. By examining the potential benefits and limitations, we aim to illuminate future directions for research and encourage the responsible integration of AI into pediatric cardiology.

## 2. Overview of AI Methodologies

Various AI methodologies have been employed in ECG analysis, each offering unique advantages and limitations. CNNs are prominent due to their ability to learn hierarchical feature representations from meticulously labeled raw input and imaging data. This allows them to be effective in classification and prediction tasks without the need for manual feature extraction of components of the ECG, such as various intervals or amplitudes [33]. CNNs have been widely used in both adult and pECG analysis. For instance, Chen et al. developed a CNN-based model called CHDdECG, which combines raw waveform features, human-concept features, and wavelet characteristics to enhance the accuracy of congenital heart disease detection [29]. This approach demonstrates how integrating diverse feature types within a CNN architecture can improve model performance.

Recurrent neural networks (RNNs) and long short-term memory networks (LSTMs) are designed for sequential data, capturing temporal dependencies in time-series signals such as ECGs [34]. Support vector machines (SVMs) and ensemble methods such as random forests have also been utilized for classification tasks, offering benefits in interpretability and performance with smaller datasets [35,36]. XGBoost (extreme gradient boosting) is another powerful ensemble learning method that has shown promise in ECG analysis [37]. XGBoost builds upon the principles of gradient boosting, creating a series of decision trees that progressively refine predictions. Due to the challenges of using ECG data, models such as SVMs or random forests do not have the same degree of accuracy as deep neural networks, and thus are not used for this purpose as often.

Adult AI-ECG research has explored innovative methodologies that could also be used in pediatrics. For example, remote monitoring technologies and wearable devices have been used to detect atrial fibrillation and other arrhythmias [31,32]. These technologies leverage DL algorithms to analyze ECG data in real-time, providing continuous monitoring and early detection of arrhythmias. Unfortunately, the minimum age of inclusion in these studies was 21 years, thus excluding children from the development, validation, and FDA clearance of these models. Applying similar technologies to pediatric populations with chronic cardiac conditions could facilitate home-based monitoring.

In hospitalized pediatric patients, ECG and other physiological data have been used to develop AI models to predict clinical deterioration. Notably, Rusin et al. have created algorithms utilizing ST segment instability for the detection of cardiopulmonary deterioration in patients with single ventricle physiology [38,39,40]. Additionally, their logistic regression models, including electrocardiographic (heart rate, heart rate variability, ST-segment elevation, and ST-segment variability) and photoplethysmography (peripheral oxygen saturation and plethysmography variability index) models, were found to be both sensitive and specific for detecting impending events 1–2 h in advance of clinical decompensation (AUROC 0.958) [39]. These predictive models require external validation and testing in randomized clinical trials to confirm their impact on outcomes with and without proactive interventions.

Each AI technique presents strengths and limitations. CNNs excel in spatial feature extraction but may require large datasets, which can be challenging in pediatric populations and rare diseases due to data scarcity. Techniques such as transfer learning and data augmentation may be able to help mitigate this issue. RNNs are adept at capturing temporal patterns but can suffer from issues such as vanishing gradients (where the learning weights that occur over time grow or shrink in value to the point where it is meaningless). SVMs are effective with smaller datasets but may be less capable of capturing the complex patterns inherent in ECG signals. Ensemble methods offer robustness and interpretability but can be computationally intensive. Selecting the appropriate AI methodology depends on the specific application, data characteristics, and clinical requirements. Recent studies have shown that integrating multiple types of features and employing techniques to enhance interpretability can improve model performance and acceptance in clinical settings [29].

Generative AI and large language models (LLMs) have become increasingly referenced in the medical literature and clinical lexicon. A recent study attempted to use an LLM (GPT-4, OpenAI) to interpret electrograms from a textbook of ECG examples, which showed higher diagnostic accuracy than emergency medicine physicians [41]. Unfortunately, this technique is fraught with limitations. LLMs are known to be prone to overfitting and these ECGs may have been a representation of common findings and would experience limitations for rarer presentations. Secondly, there is an opportunity for data leakage where the training data (the Internet in this case) have the same ECG printed elsewhere. This allows the model to “cheat” by “memorizing” data it has seen previously. Generative AI and LLMs have yet to be proven in diagnostic and prognostic prediction for ECGs, and their use remains to be studied.

## 3. Systematic Review of AI-ECG in Pediatric Cardiology

### 3.1. Methods

A miniature literature review of the literature was performed using the PubMed database. The following search was conducted using MeSH terms and filters: (“Artificial Intelligence” [MeSH Terms] AND “Electrocardiography” [MeSH Terms]) AND (allchild [Filter]). We included peer-reviewed studies published in English and indexed in PubMed. Further, the study had to include testing, validation, or a trial that applied ECG AI analysis for cardiovascular disease. Studies focusing solely on adult populations, non-ECG data studies, reviews, editorials, conference abstracts without sufficient details, and case reports or studies with fewer than 20 participants were excluded. Studies with a primary analysis of electroencephalogram or sleep studies that utilize ECG as a secondary sensor were not included.

### 3.2. Results

**Figure 1:**
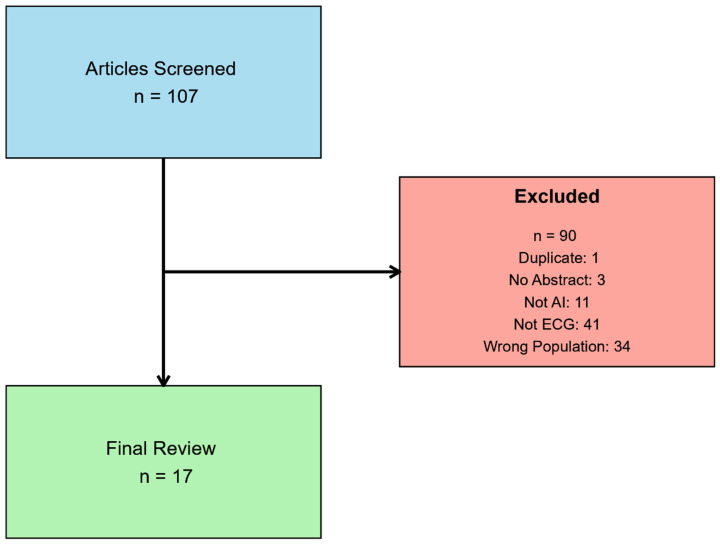
Flowchart of the systematic review process for AI-based studies in pediatric ECG analysis. The initial search identified 107 articles for screening. After evaluating each study, 90 articles were excluded based on the following criteria: duplicate records (*n* = 1), lack of an abstract (*n* = 3), studies not involving artificial intelligence (*n* = 11), studies not related to ECG (*n* = 41), and studies involving the wrong population (*n* = 34). This process resulted in 17 articles meeting the inclusion criteria for the final review.

A total of 107 studies were screened and 90 were excluded due to duplication (*n* = 1), no abstract (*n* = 3), not AI (*n* = 11), not primarily using ECG (*n* = 41), and wrong patient population (*n* = 34). This left 17 articles for review. The breakdown of this can be visualized in Figure 1 and a summary of the articles is available in Table 1.

### 3.3. Detection of Congenital Heart Diseases

Chen et al. developed a CNN-based model called CHDdECG for CHD detection using a dataset of 93,127 pediatric ECGs from three centers in China [29]. The model combines raw waveform features, human-concept features, and wavelet characteristics to enhance accuracy. It achieved an AUROC of 0.92 and a specificity of 0.881 on an internal test set of 12,000 cases, and an AUROC of 0.91 and 0.92 in two external test sets, outperforming an experienced pediatric cardiologist’s interpretation of the ECG. This study highlighted the potential of AI in identifying CHDs that may not be easily detectable through routine ECG interpretation. Limitations included the need for external validation in diverse populations and the potential for biases due to data heterogeneity.

Mori et al. designed a deep learning model trained on 728 patients to diagnose atrial septal defects (ASDs) with an AUROC of 0.95, outperforming experienced pediatric cardiologists with higher sensitivity and specificity [43]. While the study demonstrated the potential utility of AI, limitations included a small sample size and potential overfitting. Mayourian et al. similarly developed a CNN model to evaluate 46,231 ECGs for the presence of a secundum ASD [4]. They showed an internal AUROC of 0.84 with the best performance in 3–8-year-old children. This model was superior when compared to the presence of right bundle branch block alone.

De Vries et al. performed a unique study to predict congenital heart disease based on fetal ECGs [47]. Using abdominal electrodes on the mother, they filtered out maternal ECG signals and trained a neural network. The algorithm performed moderately with an AUROC of 0.76. While this study was limited due to a small sample (122 ECGs) and likely overfitting from training on multiple ECGs from the same encounter, it was an exploratory study for a novel technique in the detection of CHD that opens the possibility for early detection.

### 3.4. Arrhythmia Classification

Bos et al. used AI to detect concealed long QT syndrome in 2059 patients (both children and adults) by identifying subtle ECG features associated with increased risk [8]. The AI model achieved an AUC of 0.900 for detecting long QT syndrome and an AUC of 0.863 for identifying the condition among those with a normal resting QTc. Additionally, the model could distinguish between the three main genotypes of long QT syndrome. This study addressed a critical predictor of sudden cardiac death, though it was limited by its reliance on single-center data.

Localizing the accessory pathway in Wolff–Parkinson–White using only a 12-lead ECG poses some challenges, and accurately predicting its location helps with procedural planning prior to cardiac ablation. Nishimori et al. addressed this by training a convolutional neural network (CNN) on ECG and chest X-ray data from 1725 patients (both adults and children), achieving a pathway localization accuracy of 80% [44].

An older study completed by Edenbrandt et al. used a traditional neural network to detect limb lead reversal by computationally reversing limb amplitudes and using ECG intervals [42]. While it reported an impressive AUROC of 0.999, the small sample size (*n* = 1908) and lack of a proper holdout set likely resulted in data leakage, as the same ECGs were used to generate and test the reversal. This overestimated performance underscores the need for rigorous validation, including independent holdout sets, to ensure reliable model evaluation.

Using a separate set of modalities from those described above, Rahman et al. utilized five different algorithms, including RNNs and CNNs, to identify neonatal bradycardia in premature infants [49]. The goal was to reduce the false positive alarms that cause alarm fatigue in the neonatal intensive care unit. They were able to train a model with high sensitivity (0.992) and a low false positive rate (0.007), meeting their goals of reducing false positives by 36% over conventional methods. Given that this was only completed on 10 patients, this will need to be expanded and validated before it is ready for clinical use.

### 3.5. Detection of Ventricular Dysfunction

Mayourian et al. developed a CNN model using ECG data trained on 92,377 ECGs to predict left ventricular dysfunction, hypertrophy, and dilation in children, showing promising results for early diagnosis and management [5]. The model achieved an AUROC of greater than 0.92 on the internal and external validation training set for detecting LV dysfunction. This study also utilized a form of explainable AI (ExAI) called saliency mapping to highlight features of the ECG that were important for model performance. This can improve clinicians’ understanding of the “black box” nature of the algorithm and help them learn from the algorithm regarding various clinical pathologies. Limitations included the need for larger multicenter studies to validate findings amongst different patient demographics. Despite these excellent results, the sample size for this study was significantly smaller than that of the landmark adult studies identifying reduced ventricular function.

Their group also trained a CNN with 8584 paired ECGs and cardiac magnetic resonance (CMR) images to detect LV and RV dysfunction and dilation in patients with a known diagnosis of CHD [3]. This performed similarly to the above algorithm with an AUROC of at least 0.87 on internal and external validation to detect LV dysfunction. They found that patients with tetralogy of Fallot had the highest likelihood of LV dysfunction. They propose that this model can be used to help determine the timing of future CMR studies.

Anjewierden and O’Sullivan et al. developed pediatric models using just over 10,000 ECGs trained to predict an LVEF ≤ 35% or <50%, achieving AUC values of 0.93 and 0.88, respectively [30]. Additionally, they demonstrated a novel pediatric model for the detection of RVSD, with an AUC of 0.90. Similar to the adult algorithm mentioned above, they were able to show that those initially categorized as false positives had a 3-fold risk of developing low LVEF in the future when compared to those who were not (HR 3.34 CI, 1.94–5.77) [30].

### 3.6. Risk Stratification

Nogimori et al. used the process of transfer learning, taking an ECG-based CNN model designed to predict major cardiovascular events in adults and modifying it to estimate neurohormonal activation via elevated brain natriuretic peptide (BNP) levels [48]. It was developed using 21,378 ECG–BNP pairs from 8324 children. They then used the predicted BNP model weights to adjust the prediction for major cardiac events. This model achieved an AUROC of 0.826 for detecting major adverse cardiovascular events and is superior to the absolute BNP level alone.

A separate study by Mayourian and their group evaluated a model to predict mortality in patients with congenital heart disease based on a CNN evaluation of CHD [50]. This had a single-center AUROC of 0.97 and generated interesting hypotheses about ECG signal data and how they relate to cardiovascular function and risk. This model has not yet been externally validated. They also applied this same approach to assess mortality risk in patients with repaired tetralogy of Fallot in the INDICATOR cohort [51]. Although the AUROC of 0.81 in the external validation cohort was lower than that of the broader model, it surpassed the predictive performance of established high-risk features, including the biventricular global function index and QRS duration. Saliency mapping and ExAI identified key high-risk features such as QRS fragmentation, wide and low-amplitude QRS complexes, and flattened T waves.

### 3.7. Other Predictions

Machine learning predictions using ECG signals have also been used to detect various other conditions or situations. For instance, O’Sullivan et al. were able to build a model to predict patient age and sex with changes that occur during puberty (AUROC of 0.91) [2]. Another novel use of this technology was published by Sarkar et al. to predict fetal/maternal stress using the fetal stress index [45]. Using a “self-learning” semi-supervised CNN, they were able to predict stress with high accuracy by using data from five prior studies on the topic (AUROC of 0.982). Each individual study had fewer than 100 participants and further validation will be required for this model.

Similar to the above, other pediatric heart conditions can be detected by ECG-AI. The study by Siontis et al. utilized a CNN to predict if children have hypertrophic cardiomyopathy based on their ECG [46]. This model had excellent performance with an AUROC of 0.98 on a sample of 300 patients with hypertrophic cardiomyopathy and 18,439 controls. When younger patients (<5 years old) were tested separately, the AUROC increased to 0.99 due to lower accuracy in the younger patients, and genotype had no impact on the model.

### 3.8. Critical Analysis

The studies identified from the systematic review employed various AI techniques, predominantly deep learning models such as CNNs. Data preprocessing steps included filtering, normalizing, and segmenting ECG signals. Most studies used supervised learning approaches, requiring annotated datasets for training. Commonly reported evaluation metrics were accuracy, sensitivity, specificity, and AUROC. Cross-validation techniques and independent test sets were used to assess model generalizability.

Methodological strengths included the use of advanced AI techniques and comparative analyses with expert clinicians and human-in-the-loop analysis, demonstrating potential clinical utility. Data limitations, such as small sample sizes in some studies and a lack of diversity, may limit the generalizability of the models. Many models were not validated on external datasets, raising concerns about performance in different clinical settings. Few studies addressed the explainability of AI models, which is important for clinical acceptance.

Despite some of these limitations, these studies represent the first steps in applying the rapidly growing field of AI to benefit care for children. Once these techniques and algorithms have been properly externally validated and studied with pragmatic control trials, they hold the potential to democratize specialty knowledge and bring cheaper but accurate diagnoses to resource-limited areas. Deployment of these models in real-world areas may also help with improving diagnostic fidelity and enhancing treatment due to early diagnosis, especially in terms of the improved detection of left ventricular dysfunction.

## 4. Challenges in Pediatric ECG Interpretation

### 4.1. Age-Dependent Physiological Variations

Significant physiological changes occur as children grow, affecting ECG parameters. Heart rates are higher in infants and decrease to adult values during adolescence [52]. Changes in the size and position of the heart alter ECG waveforms, including axis orientation, interval durations, and amplitudes [53]. Age-specific reference values are necessary because normal ranges for infants differ markedly from those for teenagers. AI models must be trained on age-specific data and incorporate mechanisms to account for developmental changes to avoid misclassification and erroneous diagnoses. This challenge can be addressed by using age-stratified training datasets that include features that account for age-dependent variations.

### 4.2. Heterogeneity of Congenital Heart Disease

CHD encompasses a wide range of structural and functional heart abnormalities present from birth [54]. These conditions have diverse manifestations on ECGs. For instance, tetralogy of Fallot will have variations in the ECG that change following surgical correction [55]. Common conditions, such as coarctation of the aorta, may present with different ECG patterns, or no changes at all. Complex CHD, such as patients with situs inversus, will have a reversed electrical pattern on a standard ECG and would not be identified if not included in the AI training dataset [56]. The rarity of certain CHDs limits data availability. Some CHDs present minimal ECG abnormalities, requiring highly sensitive models. AI algorithms must identify diverse patterns and differentiate conditions with overlapping features. Chen et al. showed that integrating various feature types can improve a model’s ability to detect a wide range of CHDs [29].

### 4.3. Rarity of Pediatric Heart Disease

Congenital heart disease affects approximately 1–2% of live births and acquired and genetic heart diseases in children are relatively rare [54]. As a result, algorithms developed for these conditions must be carefully evaluated to address their unique features. Metrics such as sensitivity and specificity, which are often used to assess model performance through measures such as the AUROC, can overestimate real-world utility when the predicted condition occurs infrequently in the population. Moreover, AUROC assigns equal importance to sensitivity and specificity, which may not align with the differing priorities of medical tests—screening tests prioritize sensitivity, while confirmatory tests may focus more on specificity.

To address these limitations, researchers should also report and plot the area under the precision–recall curve (AUPRC) [57]. AUPRC highlights the precision (positive predictive value) and recall (sensitivity) of the positive class, making it particularly suitable for imbalanced datasets [58,59]. By focusing on the model’s ability to correctly identify positive cases, precision–recall curves avoid over-reliance on negative predictions to artificially boost specificity. This is exceptionally important in pediatric cardiology. Another valuable metric for imbalanced datasets is the F1 score, which represents the harmonic mean of precision and recall, providing a balanced single-value metric [60]. However, the traditional F1 score has its own limitations in scenarios where the costs of false positives and false negatives differ significantly [57]. In such cases, alternative formulations, such as weighted F1 scores or other domain-specific metrics, may better reflect the model’s real-world performance.

### 4.4. Limited Data Availability and Privacy Concerns

Compared to adult populations, pediatric ECG datasets are scarce due to ethical considerations in obtaining consent and ensuring data privacy for minors. The rarity of certain conditions and variability in ECG acquisition across institutions contribute to data heterogeneity. Small sample sizes can lead to overfitting, where models perform well on training data but poorly on unseen data. Strategies to overcome data limitations include data augmentation, transfer learning, and collaborative multi-institutional data sharing. Federated learning offers a promising solution by allowing AI models to be trained across multiple institutions without sharing raw data, enhancing privacy and data diversity [61,62]. Implementing federated learning in pediatric cardiology could improve model robustness without the risks associated with data centralization.

### 4.5. Ethical and Legal Considerations

The use of AI in pediatric healthcare raises important ethical questions. Protecting patient privacy and obtaining appropriate consent are critical considerations, especially when dealing with minors. Unrepresentative datasets can lead to algorithmic biases, affecting diagnostic equity [1]. Compliance with regulatory frameworks from bodies such as the United States FDA and the European Medicines Agency (EMA) is necessary for clinical implementation. Transparency and accountability are essential; clinicians need to understand AI decision-making processes to trust and effectively use these tools. The emergence of generative AI models and concerns about data re-identification emphasize the need for robust data governance and security measures [63,64]. In addition, machine learning techniques can potentially allow for the de-identification of subjects in certain circumstances, as they have been found to have high predictive capabilities even when the primary data have been heavily distorted and filtered [65].

### 4.6. Integration into Clinical Workflow

Adopting AI tools in clinical practice requires careful planning and execution. User-friendly interfaces that present information clearly and align with existing electronic health record (EHR) and diagnostic systems are essential [66]. Effective training programs are needed to educate clinicians on the capabilities and limitations of AI, fostering confidence and proper utilization. Importantly, AI should serve as a supportive tool to enhance, rather than replace, clinical judgment, offering decision support without dictating care. Lessons from AI-ECG applications in adult populations emphasize the critical role of interoperability and adherence to data standards for successful implementation [67].

## 5. Discussion

**Figure 2:**
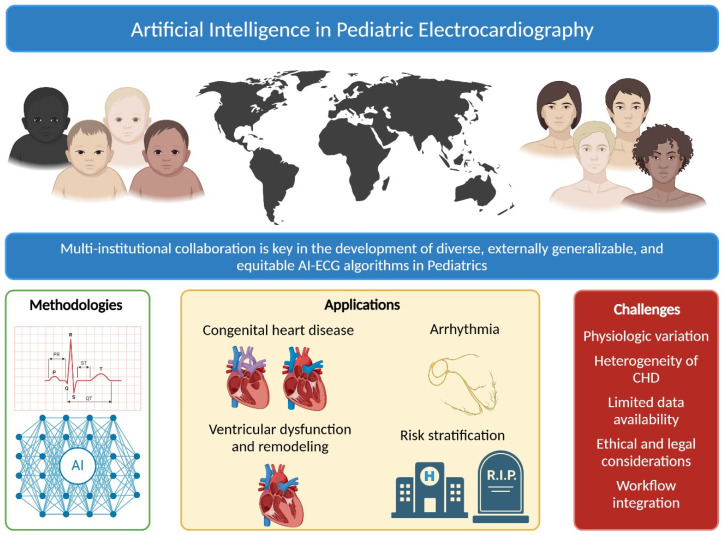
Artificial Intelligence in Pediatric Electrocardiography. AI = artificial intelligence, ECG = electrocardiogram, CHD = congenital heart disease.

AI applications in pECG analysis show significant promise in improving diagnostic accuracy, enhancing efficiency in resource-limited settings, facilitating early detection of cardiac abnormalities, and supporting clinical decision-making (Figure 2). The high sensitivity and specificity of these models make them particularly valuable for large-scale screening programs, especially in regions with limited access to pediatric cardiologists. Automated ECG analysis can prioritize children needing further evaluation, helping to optimize healthcare resource allocation. However, to ensure equitable outcomes, it is essential that AI model training and validation datasets include diverse populations to prevent the reinforcement or exacerbation of racial, ethnic, and geographic disparities in healthcare.

AI holds the potential to uncover novel ECG features linked to cardiac conditions and clinical outcomes. By leveraging large datasets, AI models can identify previously unrecognized patterns, while advancing our understanding of pediatric cardiac physiology and pathology. For example, AI models capable of predicting organ age may outperform chronological age in risk stratification, offering more precise insights for managing congenital heart disease despite its inherent heterogeneity [68].

Developing ExAI models is also imperative for clinical acceptance. When clinicians understand how AI models arrive at their conclusions, trust and effective utilization are likely to increase. Techniques such as saliency maps, layer-wise relevance propagation, and attention mechanisms can offer valuable insights into AI decision-making processes [69]. For example, the work by Mayourian et al. highlights the importance of incorporating interpretability, such as linking lateral T-wave inversion to left ventricular dysfunction [5]. While ExAI methods improve understanding, trust also depends on the external generalizability of these models and evidence from clinical trials demonstrating their efficacy and cost-effectiveness. Currently, randomized clinical trials that assess the real-world effectiveness of these models are limited. Without such validation through established standards, clinician adoption and integration into clinical practice are likely to remain limited.

Adopting AI tools in clinical settings requires seamless workflow integration, user-friendly interfaces, and adequate clinician training to support clinical judgment. Pilot studies are essential to evaluate feasibility and ensure interoperability with existing EHR systems, as tools that complicate workflows may encounter resistance. Collaborative data-sharing and federated learning can be used to increase diversity in datasets and to maintain data quality and privacy. Addressing ethical issues such as data privacy and bias is necessary for maintaining trust. Additionally, insights from adult AI-ECG applications suggest that wearable devices and remote monitoring technologies can enhance pediatric care by enabling continuous patient monitoring, early detection of cardiac issues, and improved management of chronic conditions.

### Future Directions

The future directions for AI applications in pediatric ECG analysis are promising and multifaceted. Federated learning offers a solution to data scarcity by allowing AI models to be trained across multiple institutions without sharing raw data, enhancing privacy and data diversity [62]. This approach not only enhances data diversity but also addresses confidentiality concerns, which is critical for pediatric research applications. It does come with some drawbacks in that it increases the complexity of the model, often requires a central or cloud computational architecture, and can be limited in use if there is significant heterogeneity of the datasets.

The convergence of edge (on the device) computation and wearable devices represents an exciting opportunity for real-time analysis and continuous monitoring. By processing data locally on devices, such as wearable ECG monitors, these technologies can enhance privacy and reduce latency [70]. This approach aligns with the growing trend of remote patient monitoring and could significantly improve the management of chronic cardiac conditions in children. Personalized medicine stands to benefit greatly from AI integration in pediatric cardiology. By leveraging patient-specific data and creating “digital twins”, AI can facilitate tailored treatment plans and interventions, potentially leading to improved outcomes [71,72,73]. This precision medicine approach holds particular promise in addressing the unique needs of pediatric patients with complex cardiac conditions.

AI validation and model performance studies need to be upheld to AI publishing guidelines [74,75]. Due to the relatively low incidence of heart disease in children, leading to smaller study sizes and imbalanced datasets, proper model analysis will need to be performed. This may mean a shift of focus from AUROC to AUPRC and F1 as the key measurements due to their ability to discern how well an algorithm can detect rarer events, as described above.

To validate AI models in real-world settings, large-scale prospective clinical trials are needed. These studies should assess not only diagnostic accuracy but also the impact on patient outcomes and healthcare utilization. The results from such trials will be important in establishing guidelines for clinical implementation and driving widespread adoption. Once models are initially validated, adoption into clinical practice remains a challenge. Therefore, the development of clear regulatory frameworks is paramount for the safe and effective implementation of AI technologies in pediatric cardiology.

Collaboration between developers, clinicians, and regulatory bodies will be necessary to address validation standards and perform risk assessment and post-market surveillance. Learning from regulatory experiences in adult AI-ECG applications can inform pediatric implementations and ensure patient safety.

Ethical considerations and robust data governance will remain at the forefront of AI development in this field. With the rise of generative AI and advanced analytics, ensuring data privacy and preventing misuse are critical. Implementing strong data governance frameworks and adhering to ethical standards will be essential for maintaining public trust and ensuring the responsible use of AI in pediatric cardiology. As these future directions unfold, the potential for AI to revolutionize pediatric ECG analysis and broader cardiac care becomes increasingly evident. By addressing current challenges and embracing these emerging opportunities, AI has the power to significantly enhance diagnostic capabilities, advance personalized medicine, and ultimately improve outcomes for children with heart disease.

## 6. Conclusions

Artificial intelligence holds immense potential to transform pediatric cardiology by enhancing ECG interpretation and allowing the detection of abnormalities that are hidden from human clinicians. The studies in this review demonstrate promising novel approaches in diagnosing congenital heart diseases, predicting ventricular dysfunction, and aiding in risk stratification amongst children. Our field benefits from learning from the robust body of literature being developed in the use of AI-ECG in adults, such as insights into arrhythmia detection, identification of left ventricular dysfunction, and improved risk stratification methods. Transferring this knowledge to the unique pediatric population will require the expertise and fundamental knowledge of pediatric cardiologists to lead the way, particularly their insights into pediatric-specific disease presentations and physiological differences.

Among the neural network architectures evaluated in this study, CNNs demonstrated the greatest promise for pediatric ECG interpretation due to their robust ability to capture spatial hierarchies in waveform data. LSTMs and RNNs showed strength in modeling temporal dependencies but were more variable in performance. Generative AI shows promise due to its ability to examine multi-modal data, but this application with raw ECG data has not yet been fully studied.

Despite these strengths, limitations such as low specificity and higher false positive rates remain of concern. Metrics such as precision, recall, PPV, and NPV are particularly valuable in understanding the clinical implications of these limitations, as they directly relate to diagnostic accuracy and the impact on patient care. Future research should focus on improving specificity without compromising sensitivity to maximize clinical utility.

Realizing these gains will require addressing challenges related to smaller datasets, difficulty in model interpretability, ethical considerations, and clinical integration. Future efforts should focus on collaborative data sharing, addressing challenges related to data-sharing restrictions, developing explainable AI models that overcome interpretability shortcomings, adhering to ethical standards, and rigorous validation through prospective clinical trials. By fostering dedicated cross-disciplinary teams involving clinicians, data scientists, and ethicists, AI-ECG can become a valuable tool in improving outcomes for children with cardiovascular disease. The power of collaboration and teamwork is necessary to overcome these challenges, inspire innovation, and ensure that every child receives the best possible care. The integration of AI into pediatric cardiology promises enhanced diagnostic capabilities. Additionally, it supports the advancement of personalized medicine and equitable healthcare delivery. Successful AI integration has the potential to fundamentally transform pediatric healthcare, setting a new standard for how we diagnose, treat, and manage cardiovascular diseases in children, ultimately shaping a future where every child receives the most advanced and individualized care possible.

## Figures and Tables

**Table 1:** Summary of AI Applications in Pediatric ECG Analysis.

Description	Primary Author(s)	Year	Title	Model Type	Data Type	*n*	Outcome Metric	Main Findings
Detection of limb lead reversal [42]	Edenbrandt L	1998	Recognition of lead reversals in pediatric electrocardiograms	ANN	ECG intervals	1908 patients	Internal AUROC: 0.999	AI can detect limb lead reversals in pediatric ECGs with high accuracy *.
Detection of long QT syndrome [8]	Bos JM	2021	Use of Artificial Intelligence and Deep Neural Networks in Evaluation of Patients with Electrocardiographically Concealed Long QT Syndrome from the Surface 12-Lead Electrocardiogram	CNN	12-lead ECG	2059 patients in total ^†^	Internal AUROC 0.90 (95% CI, 0.88–0.93), F1 0.84	Detection of concealed long QT syndrome from ECG data with high accuracy.
Detection of atrial septal defects [43]	Mori H	2021	Diagnosing Atrial Septal Defect from Electrocardiogram with Deep Learning	CNN and LTSMs	ECG data	728 patients	Internal AUROC: 0.95, F1 0.81	Detection of atrial septal defect from ECGs with high accuracy.
Multimodal location of accessory pathways [44]	Nishimori M	2021	Accessory pathway analysis using a multimodal deep learning model	CNN	12 lead ECG and chest X-ray images	294 patients with WPW and 1519 controls	Mean Accuracy: 0.80, F1 0.88	AI combines ECGs and CXR to locate accessory pathways in WPW syndrome with good accuracy.
Detection of maternal/fetal stress [45]	Sarkar P	2021	Detection of maternal and fetal stress from the electrocardiogram with self-supervised representation learning	Self-supervised Deep Learning	Maternal -fetus dyads using maternal and maternal abdominal ECGs	Multiple datasets, 210 total	External AUROC: 0.98, specificity 0.982	AI detects stress levels from maternal and fetal ECGs with high accuracy.
Detection of hypertrophic cardiomyopathy [46]	Siontis KC	2021	Detection of hypertrophic cardiomyopathy by an artificial intelligence electrocardiogram in children and adolescents	AI ECG analysis	12-lead ECG data from children and adolescents	300 patients	Internal AUROC: 0.98, specificity 0.95	Detection of hypertrophic cardiomyopathy using AI-ECG with high accuracy.
Detection of CHD using fetal ECG [47]	de Vries IR	2023	Fetal electrocardiography and artificial intelligence for prenatal detection of congenital heart disease	CNN	Fetal (abdominal) ECG data	122 patients	Internal AUROC: 0.76–0.78	Fetal AI-ECG can help detect CHD prenatally with fair accuracy.
Detection of secundum atrial septal defects [4]	Mayourian J	2023	Pediatric ECG-AI to Predict Secundum Atrial Septal Defects	CNN	Pediatric ECG data	46,261 patients	Internal AUROC: 0.84, AUPRC: 0.46	AI predicts secundum ASD using ECG data with good accuracy.
Prediction of sex across pediatric ages [2]	O’Sullivan D	2023	Pediatric sex estimation using AI-enabled ECG analysis: influence of pubertal development	CNN	Pediatric ECG data	90,133 patients	Internal AUROC: 0.91, specificity 0.83	Sex estimation based on AI-ECG with a higher discriminatory ability after puberty.
Detection of biventricular dysfunction [30]	Anjewierden S & O’Sullivan D	2023	Detection of Right and Left Ventricular Dysfunction in Pediatric Patients Using Artificial Intelligence–Enabled ECGs	CNN	Pediatric ECG data	10,142 ECGs	Internal LVSD: AUROC 0.93, specificity 0.89 RVSD: AUROC 0.90, specificity 0.80	Detection of LV/RVSD in pediatric patients using ECG data.
Detection of CHD [29]	Chen J	2024	Congenital heart disease detection by pediatric electrocardiogram based deep learning integrated with human concepts	CNN integrated with wavelet transformations	9-lead ECG	65,869 in the training set, 12,000, 7137 and 8121 in internal/external test set.	External AUROC: 0.907–0.917 Specificity: 0.907–0.937	Integrating human concepts improves CHD detection with high accuracy.
Detection of LV dysfunction, dilation, and hypertrophy [5]	Mayourian J	2024	Pediatric ECG-Based Deep Learning to Predict Left Ventricular Dysfunction and Remodeling	CNN	12-lead ECG	92,377 ECGs in training set, 12,631; 2830 and 5088 in internal/external test set.	LVSD AUROC: 0.94 AUPRC: 0.32 LV dilation AUROC: 0.87 AUPRC: 0.33 LVH AUROC: 0.84 AUPRC: 0.25	Prediction of left ventricular dysfunction and remodeling with high accuracy.
Prediction of biventricular dysfunction in patients with CHD [3]	Mayourian J	2024	Deep Learning-Based Electrocardiogram Analysis Predicts Biventricular Dysfunction and Dilation in Congenital Heart Disease	CNN	12-lead ECG	8584 ECGs ^†^	External AUROC: LVSD: 0.89 LV dilation: 0.83 RVSD: 0.82 RV dilation: 0.80	Prediction of biventricular dysfunction and dilation in CHD patients with good accuracy.
Prediction of MACE using ECG and BNP [48]	Nogimori Y	2024	Prediction of adverse cardiovascular events in children using artificial intelligence-based electrocardiogram	CNN	12-lead ECG data and BNP levels	8324 ECGs	AUROC: 0.826 (95% CI, 0.706–0.945), specificity 0.655	Prediction of MACE using ECG and BNP with good accuracy.
Detection of neonatal bradycardia [49]	Rahman J	2024	Machine learning model with output correction: Towards reliable bradycardia detection in neonates	RNN, LSTM, CNN	440 h real time 3-Lead ECG data	10 patients	Internal AUROC CNN + LSTM: 0.987, AUPRC 0.73	Improved detection of bradycardia in premature infants, reducing false positive alarms.
Prediction of mortality among patients with CHD [50]	Mayourian J	2024	Electrocardiogram-based deep learning to predict mortality in paediatric and adult congenital heart disease	CNN	12-lead ECG	39,784 patients ^†^	Internal AUROC: 0.79, AUPRC 0.17	Mortality prediction in pediatric and adult CHD patients with high accuracy.
Prediction of mortality in repaired TOF [51]	Mayourian J	2024	Electrocardiogram-Based Deep Learning to Predict Mortality in Repaired Tetralogy of Fallot	CNN	12-lead ECG	78,578 patients	External AUROC: 0.81, AUPRC 0.21	Mortality prediction in patients with repaired TOF with good accuracy.

* This paper demonstrates some flaws in the methods, including the lack of a holdout set that likely led to the high AUROC. ^†^ Number of pediatric patients not documented. AI = artificial intelligence, ANN = artificial neural network, AUROC = area under receiver operator curve, BNP = B-type natriuretic peptide, CHD = congenital heart disease, CNN = convolutional neural network, CXR = chest X-ray, ECG = electrocardiogram, LSTM = Long short-term memory, LVH = left ventricular hypertrophy, LVSD = left ventricular systolic dysfunction, MACE = major adverse cardiac events, RNN = recurrent neural network, RVSD = right ventricular systolic dysfunction, WPW = Wolff–Parkinson–White.

## Data Availability

No new data were created or analyzed in this study. Data sharing is not applicable to this article.

## Abbreviations

AI	Artificial Intelligence
AUROC	Area Under the Receiver Operating Curve
AUPRC	Area Under the Precision Recall Curve
CHD	Congenital Heart Disease
CNN	Convolutional Neural Network
DL	Deep Learning
ECG	Electrocardiogram
ExAI	Explainable Artificial Intelligence
FDA	Food and Drug Administration
LSTM	Long Short-Term Memory Networks
ML	Machine Learning
pECG	Pediatric Electrocardiogram
RNN	Recurrent Neural Networks
SVM	Support Vector Machines
